# Reduced graphene oxide-loaded nanocomposite scaffolds for enhancing angiogenesis in tissue engineering applications

**DOI:** 10.1098/rsos.172017

**Published:** 2018-05-02

**Authors:** S. Chakraborty, T. Ponrasu, S. Chandel, M. Dixit, V. Muthuvijayan

**Affiliations:** Department of Biotechnology, Bhupat and Jyoti Mehta School of Biosciences, Indian Institute of Technology Madras, Chennai 600036, Tamil Nadu, India

**Keywords:** tissue engineering, reduced graphene oxide, nanocomposite scaffolds, angiogenesis, vascularization

## Abstract

Tissue engineering combines cells, scaffolds and signalling molecules to synthesize tissues *in vitro*. However, the lack of a functioning vascular network severely limits the effective size of a tissue-engineered construct. In this work, we have assessed the potential of reduced graphene oxide (rGO), a non-protein pro-angiogenic moiety, for enhancing angiogenesis in tissue engineering applications. Polyvinyl alcohol/carboxymethyl cellulose (PVA/CMC) scaffolds loaded with different concentrations of rGO nanoparticles were synthesized via lyophilization. Characterization of these scaffolds showed that the rGO-loaded scaffolds retained the thermal and physical properties (swelling, porosity and *in vitro* biodegradation) of pure PVA/CMC scaffolds. *In vitro* cytotoxicity studies, using three different cell lines, confirmed that the scaffolds are biocompatible. The scaffolds containing 0.005 and 0.0075% rGO enhanced the proliferation of endothelial cells (EA.hy926) *in vitro*. *In vivo* studies using the chick chorioallantoic membrane model showed that the presence of rGO in the PVA/CMC scaffolds significantly enhanced angiogenesis and arteriogenesis.

## 1. Introduction

Tissue engineering is a multidisciplinary field that aims at creating tissues *in vitro*. In nature, biological tissues consist of different cell types, each with a particular function, embedded within the extracellular matrix. These cells require a continuous supply of oxygen and nutrients to survive and function optimally. The vascular system supplies these tissues with oxygen and nutrients. It also helps in the removal of carbon dioxide and other wastes. Hence, it is imperative that any artificial tissues created *in vitro* have vascular-like structures or are designed such that they lead to quick vascularization upon implantation. Although various strategies for incorporating a vascular network in artificial tissue constructs have been explored, vascularization still remains a major limitation that causes failure of engineered tissue *in vivo* [1,2]. As a result, many tissue-engineered constructs that have been shown to be effective *in vitro* fail *in vivo* [1,3]. Vascularization of a tissue-engineered construct is a complex phenomenon, involving contributions from the scaffold (microarchitecture and stiffness) and pro-angiogenic moieties (growth factors, small molecules and nanoparticles). Lack of vascularization exposes the cells implanted within the engineered construct to hypoxia, thus causing a lethal environment for the cells [4]. Lack of efficient techniques for vascularization limits the size of tissue-engineered three-dimensional constructs [2,5]. The requirement of a mature vasculature has been repeatedly demonstrated in different tissue engineering applications [6–9].

It has also been demonstrated that maturation of the vascular network formed within the tissue-engineered scaffold is crucial to the success of the construct *in vivo* [10]. Blood vessel maturation is a complex phenomenon requiring the participation of different growth factors and cell types. Current approaches for overcoming the problem of vascularization can be broadly classified into three categories: biochemical approaches [10–12], cell-based approaches [13–15] and micro-engineering techniques [16–18]. In the biochemical approaches, the scaffold is loaded with growth factors or small bioactive molecules that are involved in vascularization *in vivo*. In the cell-based approaches, mature or progenitor endothelial cells are co-cultured along with the cells of interest within the scaffold, with the expectation that this will lead to the formation of vascular networks *in vivo*. In the micro-engineering approaches, techniques typically used in semiconductor industries for modification of polymers are used to form vascular-like structures within the scaffolds. However, all of these techniques have their own limitations, such as the quick degradation of the growth factors in physiological conditions due to their short half-lives in the biochemical approach, lack of control over differentiation of the seeded cells and dissociation of the cells in the cell-based approach, and lack of host integration in the micro-engineering approach [1,2].

Vascular endothelial growth factor (VEGF) has been used extensively as a pro-angiogenic protein in tissue engineering applications [2]. Despite some success, the lack of spatio-temporal control over the release of such protein growth factors has led to complications such as leaky vasculature [19]. Also, the short half-lives of such proteins in physiological conditions have led to some scepticism about the feasibility of using them as a therapeutic agent. In the recent years, there is a growing interest in using graphene-based materials for tissue engineering and other biomedical applications [20,21]. Graphene and reduced graphene oxide (rGO) are good conductors of electricity and have thus been used for neural tissue engineering applications [20,22,23]. Graphene, graphene oxide (GO) and rGO have also been used in cardiac, cartilage and optical tissue engineering due to their excellent mechanical and optical properties [20,24–30]. In a recent study, rGO nanoparticles were shown to be pro-angiogenic up to a certain concentration, when used in a solution [31]. The pro-angiogenic behaviour of rGO was attributed to its ability to increase the intracellular concentration of reactive oxygen species [31]. In this work, we have studied the angiogenic potential of rGO loaded to a tissue-engineered scaffold by preparing rGO loaded to polyvinyl alcohol/carboxymethyl cellulose (PVA/CMC) nanocomposite hydrogels.

Hydrogels have been used extensively for tissue engineering applications due to their tuneable mechanical properties and ease of fabrication. Their high water retention ability, which allows nutrients, growth factors and waste products to diffuse through them, makes them suitable substrates for mammalian cell growth and differentiation. Based on the source of the polymers used in fabricating the hydrogels, they fall under three broad categories: natural, artificial and natural–artificial composites. Among the various artificial polymers that have been successfully used to synthesize hydrogels for tissue engineering, PVA has good biocompatibility and mechanical strength [32]. It is also amenable to various chemical and physical modifications. Thus, it is suitable for use in conjugation with other polymers [32]. However, due to its excessive hydrophilic nature, significant attachment of mammalian cells on PVA surfaces is not achieved [33]. This property of a hydrogel is crucial in determining its applicability as a scaffold for tissue engineering applications. Thus, to enhance cell attachment on PVA hydrogels, it is usually conjugated with a natural polymer or a derivative of a natural polymer. Alginate, cellulose, ovalbumin, dextran, heparin, gelatin, hyaluronic acid, chondroitin sulfate, chitosan, silk and starch have all been blended with PVA for various biomedical applications [34]. Among the naturally derived polymers routinely conjugated with PVA, CMC, which is a carboxymethyl derivative of the natural polymer cellulose, is biocompatible and allows cell attachment, growth and migration [35]. Hence, in this work, we have prepared PVA/CMC hydrogels as the model scaffold.

Here, we report the effects of rGO incorporation into PVA/CMC scaffolds on their physical, chemical and biological properties, and the angiogenic potential of such scaffolds.

## 2. Materials and methods

### 2.1. Materials

PVA, CMC and resazurin sodium salt (alamarBlue reagent) were obtained from HiMedia Laboratories Pvt Ltd. All chemicals and reagents used were of analytical grade.

### 2.2. Methods

#### 2.2.1. Synthesis of reduced graphene oxide nanoparticles

GO was synthesized from graphite via a modified Hummers' method [36]. Briefly, 23 ml of sulfuric acid was added to a mixture of 1 g of graphite powder and 0.5 g of sodium nitrate and stirred for 30 min. The temperature of the reaction mixture was kept below 20°C through the addition of potassium permanganate. After the reaction, the mixture was stirred overnight at 35°C. The temperature was then raised to 98°C by the addition of distilled water. After several washes with 5% hydrochloric acid, the solid product was separated by filtration and dried under vacuum at 50°C. The synthesized GO had the appearance of a fine black powder. This GO was reduced under sunlight using a convex lens to obtain rGO nanoparticles [37,38]. Briefly, sunlight was focused on GO using the lens for 15 min, such that power of 1.8–2.5 W and a temperature above 300°C were reached. Exfoliation of GO into graphene sheets occurred and reduction to rGO was also achieved at this temperature. The rGO nanoparticles synthesized by the above technique were dispersed in distilled water and sonicated in a sonication bath for 30 min before any further processing. FT-Raman spectroscopy was performed on the GO and rGO nanoparticles to confirm the reduction of GO. A dynamic light scattering (DLS) technique was used to determine the average size of the synthesized nanoparticles. TEM images were also taken to observe these nanoparticles.

#### 2.2.2. Fabrication of polyvinyl alcohol/carboxymethyl cellulose/reduced graphene oxide composite hydrogel scaffolds

The scaffolds were synthesized using lyophilization to achieve a porous structure that has a high surface area, which can aid in cell penetration and release of rGO. These factors could play a critical role in accelerated angiogenesis of the engineered tissues. Briefly, a solution was prepared by autoclaving 100 ml of distilled water containing 9 g of PVA and 1 g of CMC at a temperature of 121°C and a pressure of 1034 mbar for 30 min. Simultaneously, rGO nanoparticles were dispersed in distilled water at four different concentrations (0.0025, 0.005, 0.0075 and 0.01% (w/v)). After sonication for 30 min, the rGO containing dispersion was added to the PVA/CMC solution, thus forming PVA/CMC composites with five different concentrations of rGO: PVA/CMC rGO 0% (to be used as a control and will henceforth be mentioned as just PVA/CMC), PVA/CMC rGO 0.0025%, PVA/CMC rGO 0.005%, PVA/CMC rGO 0.0075% and PVA/CMC rGO 0.01%. The solutions were then vortexed for approximately 15 s and kept at room temperature for 15 min. They were then frozen at −20°C for 30 min. This was an intermediate cooling step. After this, the scaffolds were lyophilized at −110°C and a pressure of 0.01 mbar. The conditions of temperature and pressure were maintained for 24 h. The lyophilized scaffolds were then brought back to room temperature. The lyophilized scaffolds were then used for further studies. Scaffolds were prepared either in Petri plates or tissue culture plates based on the requirement of the experiments performed on them.

### 2.3. Scaffold characterization

#### 2.3.1. FT-Raman spectroscopy

The synthesized scaffolds were analysed via FT-Raman spectroscopy for qualitative confirmation of the rGO incorporation into the scaffolds.

#### 2.3.2. X-ray diffraction analysis

To corroborate the results obtained via FT-Raman spectroscopy and to determine the effect of rGO incorporation on the crystallinity of PVA/CMC scaffolds, X-ray diffraction (XRD) analysis was performed. XRD patterns of the scaffolds were recorded in the 2*Θ* range of 5–50°. The degree of crystallinity of the scaffolds was obtained using the following formula [39]:

2.1 $$\text{Degree of crystallinity}=\frac{A_{\mathrm{cr}}}{A_{\mathrm{cr}}+A_{\mathrm{am}}}\times 100,$$

where *A*_cr_ is the area under the crystalline peak of the XRD graph and *A*_am_ is the area under the amorphous portion.

#### 2.3.3. Scanning electron microscopy

The surface morphology of the synthesized scaffolds was studied using scanning electron microscopy (SEM) imaging.

#### 2.3.4. Porosity

Scaffold porosity was measured by the hexane displacement method [40]. Briefly, scaffolds were cut into smaller cylindrical pieces of approximately equal dimensions. Their diameter and height were measured using a screw gauge and subsequently, their volumes were determined. Scaffolds were then immersed in a solution of *n*-hexane for 30 min in order for the hexane to occupy the pores within the scaffolds. Scaffolds were then removed from hexane and weighed. The percentage porosity of the scaffolds was determined using the formula:

2.2 $$\text{Porosity}\;(\%)=\frac{W_{\mathrm{WS}}-W_{\mathrm{DS}}}{\rho \times V_{S}}\times 100,$$

where *W*_DS_ is the weight of scaffold before immersion in *n*-hexane and *W*_WS_ is the weight of scaffold after 30 min of incubation in *n*-hexane, *ρ* is the density of *n*-hexane and *V*_S_ is the volume of the scaffold. These experiments were performed in triplicates and repeated thrice.

#### 2.3.5. Thermogravimetric analysis and differential scanning calorimetry

Thermogravimetric analysis (TGA) was performed to evaluate the thermal stability of the scaffolds. Furthermore, the glass transition (*T*_G_) and the melting temperature (*T*_M_) were evaluated using differential scanning calorimetry (DSC).

#### 2.3.6. Swelling

Swelling kinetics was studied to determine the degree of swelling of the scaffolds at equilibrium [41,42]. Briefly, scaffolds, cut into smaller pieces of same weight and roughly the same volume, were immersed in PBS. Samples were then removed from PBS at different time points. The moisture adsorbed on the surface of the scaffolds was removed using a tissue paper, and then the scaffolds were weighed. The swelling ratio was calculated using the following formula:

2.3 $$\text{Swelling ratio}=\frac{\left(S_{W}-S_{D}\right)}{S_{D}},$$

where *S*_D_ is the sample dry weight and *S*_W_ is the sample wet weight. These experiments were performed in triplicates and repeated thrice.

#### 2.3.7. *In vitro* biodegradation

*In vitro* biodegradation assay was performed to evaluate the rate of biodegradation of the scaffolds [41]. Briefly, scaffolds were cut into smaller pieces of equal weight and roughly equal dimensions and immersed in a 1 mg ml^−1^ lysozyme solution [43]. Scaffolds were removed from the solution after incubation for 7, 14, 21 and 30 days, dried and then lyophilized. The lyophilized scaffolds were then weighed. The percentage reduction in mass of the scaffolds at each time point was measured using the following formula:

2.4 $$\text{ Percentage reduction in mass}=-\frac{W_{T}-W_{I}}{W_{I}}\times 100,$$

where *W*_I_ is the initial weight of the scaffolds and *W*_T_ is the weight after *T* days incubation in the lysozyme solution (*T* = 7, 14, 21 and 30 days). These experiments were performed in triplicates and repeated thrice.

#### 2.3.8. *In vitro* cytotoxicity

The *in vitro* cytotoxicity of the scaffolds was studied using three different cell types, namely fibroblasts (NIH3T3), endothelial-like cells (ECV304) and endothelial cells (EA.hy926). Prior to experiments, all the cells were maintained in 60 mm tissue culture dishes in Dulbecco's Modified Eagle's Medium (DMEM) supplemented with 10% fetal bovine serum (FBS). Scaffolds were prepared in 24-well tissue culture plates and sterilized by exposure to UV light overnight in a laminar airflow hood. One day prior to the cell attachment and viability experiments, the cells maintained in tissue culture dishes were drained of their growth media, washed twice with PBS and then trypsinized. The trypsinized cell mass was then resuspended in 1 ml of fresh DMEM/10% FBS medium and centrifuged at 2000 rpm for 3 min. The supernatant was discarded and the pellet was resuspended in fresh 1 ml of DMEM/10% FBS medium. Cells were then seeded on the UV-sterilized scaffolds. After culturing the cells for 24 h, the following experiments were performed.

##### 2.3.8.1. Cell viability studies

Quantitatively, the cell viability after 24 h of culturing on the scaffolds was measured using the alamarBlue assay [44]. Briefly, cells were drained of their media and washed twice with PBS. Then, they were incubated in DMEM basal media supplemented with 0.1 mg ml^−1^ resazurin reagent for 30 min. After the incubation, the absorbance of the media was measured at 570 and 595 nm [44]. The cell viability was obtained using the formula:

2.5 $$\text{CV}\;(\%)=\frac{\left(\text{Ab}{\text{s}}_{570}E-\text{Ab}{\text{s}}_{570}B)-(\text{Ab}{\text{s}}_{570}E-\text{Ab}{\text{s}}_{570}B\right)}{\left(\text{Ab}{\text{s}}_{595}C-\text{Ab}{\text{s}}_{595}B)-(\text{Ab}{\text{s}}_{595}C-\text{Ab}{\text{s}}_{595}B\right)},$$

where CV is the cell viability, *E* is the experimental wells (the ones with scaffolds and cells), *B* is the blank (the ones with scaffolds but no cells) and *C* is the control (cells seeded on tissue culture plate). The values obtained for cell viability in the different scaffolds were normalized to that obtained in the control plate. The final value was represented as fold cell viability. These experiments were performed in triplicates and repeated six times.

##### 2.3.8.2. Cell morphology, nuclear integrity and cell attachment

Morphology of the cells cultured on the scaffolds was observed under a phase-contrast light microscope. The nuclear integrity of the cultured cells was evaluated using 4′,6-diamidino-2-phenylindole (DAPI) staining. The cells stained with DAPI were visualized under UV light in a fluorescence microscope (Olympus I5X1). The attachment of EA.hy926 cells was observed under a scanning electron microscope. For obtaining these images, cells that were cultured for 24 h were fixed onto the scaffolds using a 4% (v/v) formaldehyde solution for 10 min at 37°C. The scaffolds were then vacuum-dried and imaged under a scanning electron microscope. The voltage was maintained at 5 kV. The viability of EA.hy926 cells was analysed qualitatively using fluorescein diacetate (FDA) stain. Briefly, the media were removed after 24 h of cell culture. The scaffolds were then washed with PBS thrice and subsequently incubated for 5 min in DMEM basal media containing 0.32 mg ml^−1^ FDA. After incubation, the FDA-containing media were discarded and the cells were again washed thrice with PBS. The FDA-stained cells were then observed under a fluorescent microscope (Olympus 1X51).

##### 2.3.8.3. *In vitro* endothelial cell proliferation

The proliferation of endothelial cells (EA.hy926) was studied. EA.hy926 cells were cultured on the scaffolds for 72 h to assess the rate of proliferation of these cells on these scaffolds. Briefly, the scaffolds were UV-sterilized and then used as the platform for the proliferation of cells. AlamarBlue assays were performed after 24, 48 and 72 h of cell growth. The results were compared to determine the relative viability of the cells on different scaffolds at each time. These experiments were performed in triplicates and repeated six times.

### 2.4. Chick chorioallantoic membrane assay

To determine the suitability of the synthesized scaffolds for enhancing angiogenesis in tissue engineering scaffolds, chick chorioallantoic membrane (CAM) assay was performed. Briefly, fertilized chicken eggs at day 3 of embryonic development were drained of 3 ml albumen. This was done by drilling a very small hole on the eggshell at the region opposite to the location of the embryo and then using a syringe to drain the albumen. The eggs were then incubated in a 37°C incubator for 5 days. Then, on day 8 of embryonic development, a window was created on the eggshell above the embryo. Scaffolds cut into small circular discs were UV-sterilized overnight and then placed on top of the CAM. Sterile filter paper discs were used as an untreated control. The areas surrounding the implanted scaffolds were imaged from multiple angles. The window was then sealed with parafilm and a sterile scotch tape. Then, on day 10 of embryonic development, the window was opened again and the areas surrounding the implanted scaffolds were imaged. The images at days 8 and 10 were then analysed using AngioQuant v. 1.33. The number of blood vessels and the average thickness of the blood vessels were quantified. The data obtained for the synthesized scaffolds were normalized to those of the control at day 8. These experiments were performed in triplicates and repeated thrice.

### 2.5. Statistics

All the results are expressed as a mean ± standard deviation. One-way analysis of variance followed by Tukey's post hoc test was performed to determine the statistical significance of the data obtained for each individual scaffold. The *p-*values *p < 0.05, **p < 0.01, ***p < 0.001 were considered statistically significant.

## 3. Results

### 3.1. Synthesis and characterization of reduced graphene oxide nanoparticles

**Figure 1.**
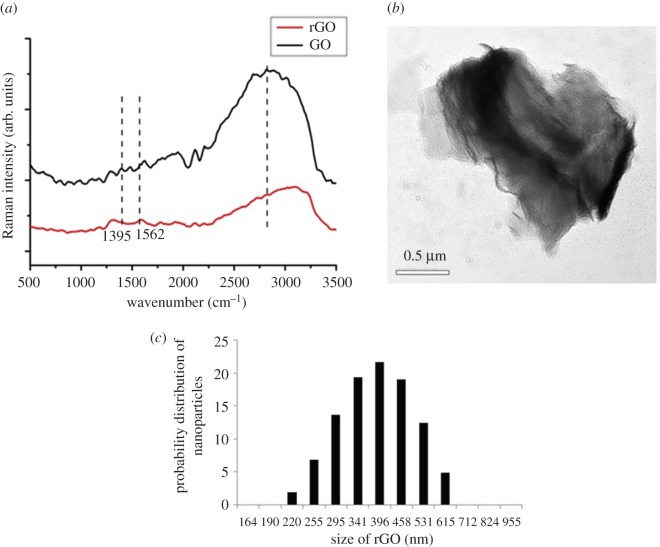
(*a*) FT-Raman spectra of GO and rGO, (*b*) TEM micrograph and (*c*) particle-size distribution of rGO.

FT-Raman spectroscopic analysis of the GO and rGO revealed two characteristic bands corresponding to graphitic materials, the G and D bands (figure 1*a*). Pristine graphite exhibits the G and D bands at 1580 cm^–1^ and 1350 cm^–1^, respectively [45]. GO that was synthesized using Hummers' method exhibited G and D bands at 1562 and 1302 cm^–1^, respectively. rGO synthesized from GO via irradiation under sunlight exhibited the characteristic G and D bands at 1584 cm^–1^ and 1316 cm^–1^, respectively. The G band, which is characteristic of all sp^2^ hybridized carbon atoms, is a consequence of first-order Raman scattering of the E_2_g vibrational mode in graphite sheets [45]. The D band occurs due to perturbations in the sp^2^ hybridization state of carbon and accumulation of structural defects. The shift in G band in the case of GO is due to a change in the hybridization state of carbon from sp^2^ to sp^3^ as a result of oxidation of graphite [45]. The shift in D band is due to the formation of structural defects as a result of a reduction in the size of sp^2^ domains. Upon reduction of GO to rGO, a shift in the G band to 1584 cm^–1^ and a shift in the D band to 1316 cm^–1^ were observed due to the restoration of the sp^2^ nature of hybridization of carbon atoms and reduction in structural defects as a consequence. Apart from the location of the bands, the ratio of intensities of the D and G bands (*I*_D_/*I*_G_) gives an indication of the degree of defects in the structure. Graphite has a very low *I*_D_/*I*_G_ ratio (0.7). GO exhibited an *I*_D_/*I*_G_ ratio of 0.9, while rGO exhibited an *I*_D_/*I*_G_ ratio of 1.1. These values further confirmed the reduction of GO to rGO.

rGO nanoparticles appeared as thin sheets under TEM (figure 1*b*). DLS analysis of the synthesized rGO nanoparticles gave a mean size of 400 nm with all particles being within the range of 200–600 nm (figure 1*c*). The size of the nanoparticles observed through TEM was approximately 500 nm, which was in agreement with the DLS data.

### 3.2. Synthesis and characterization of PVA/CMC rGO composite scaffolds

**Figure 2.**
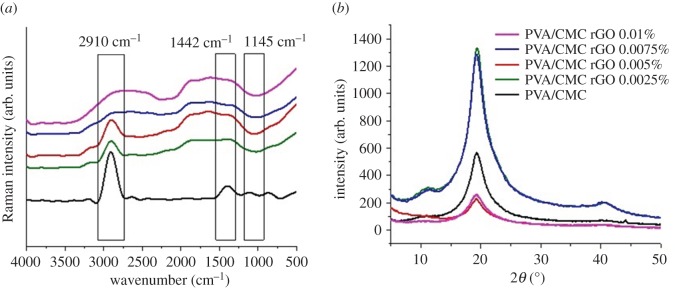
(*a*) FT-Raman spectra and (*b*) XRD patterns of PVA/CMC, PVA/CMC rGO 0.0025%, PVA/CMC rGO 0.005%, PVA/CMC rGO 0.0075% and PVA/CMC rGO 0.01%.

FT-Raman spectra of pure PVA/CMC blend exhibited characteristic peaks at 2910 and 1145 cm^–1^ [46] (figure 2*a*). After incorporation of rGO into the scaffolds, this peak shifted slightly towards the right owing to weak interactions between the functional groups in rGO and the polymer, thus confirming the incorporation of rGO into PVA/CMC blend.

XRD analysis of PVA/CMC and PVA/CMC scaffolds incorporated with varying concentrations of rGO showed a prominent peak at a 2*θ* angle of 20° (figure 2*b*), characteristic of PVA/CMC blend [47]. The intensity and the area under the peak at this particular angle kept decreasing with increasing concentrations of rGO, thus indicating a reduction in the crystalline nature of the scaffolds upon rGO incorporation. The degree of crystallinity for the PVA/CMC scaffold was taken as 100%, and the degrees of crystallinity for other scaffolds were normalized to that of the PVA/CMC scaffold (table 1).

**Table 1.** Estimated degrees of crystallinity of the scaffolds normalized against that of PVA/CMC scaffolds.

sample	degree of crystallinity
PVA/CMC	100
PVA/CMC rGO 0.0025%	87.3
PVA/CMC rGO 0.0005%	79.2
PVA/CMC rGO 0.0075%	72
PVA/CMC rGO 0.0100%	70.4

### 3.3. Physical properties of the synthesized scaffolds

#### 3.3.1. Scanning electron microscopy and porosity

**Figure 3.**
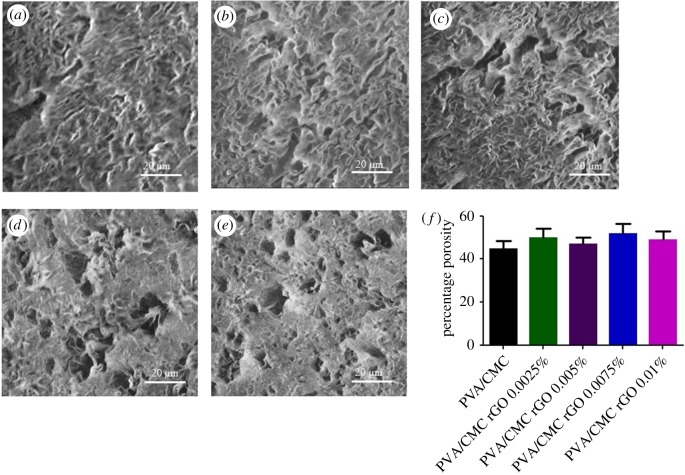
SEM micrographs (*a*) PVA/CMC, (*b*) PVA/CMC rGO 0.0025%, (*c*) PVA/CMC rGO 0.005%, (*d*) PVA/CMC rGO 0.0075%, (*e*) PVA/CMC rGO 0.01% and (*f*) porosity values of the scaffolds.

SEM images of the scaffolds revealed the porous nature of the scaffold surfaces (figure 3*a–e*). The pores were scattered throughout the surface of each scaffold and quite visibly were not of the same size. The average porosity value of the scaffolds, as obtained via the hexane displacement method, was approximately 50% (figure 3*f*). The difference in porosity among the scaffolds was not found to be significant. These observations clearly show that rGO incorporation does not alter the porosity of the scaffolds.

#### 3.3.2. Thermal properties

**Figure 4.**
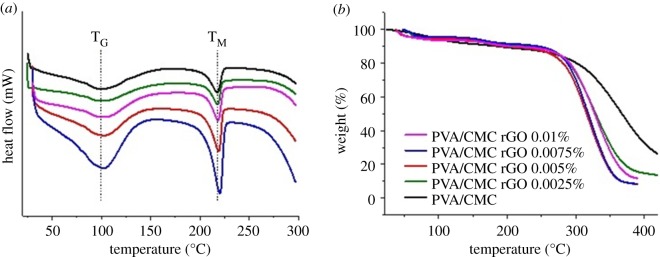
(*a*) DSC and (*b*) TGA thermograms of PVA/CMC, PVA/CMC rGO 0.0025%, PVA/CMC rGO 0.005%, PVA/CMC rGO 0.0075% and PVA/CMC rGO 0.01%.

DSC data revealed the glass transition temperature (*T*_G_) of the scaffolds to be approximately 100°C and the melting temperature (*T*_M_) to be approximately 218°C (figure 4*a*). All the scaffolds had similar values for *T*_G_ and *T*_M_. Thus, incorporation of rGO did not have any significant effect on these parameters. TGA analysis revealed the temperature at which scaffolds started losing mass was approximately 300°C (figure 4*b*). Beyond this temperature, there was a steep decrease in the mass of the scaffolds. Incorporation of rGO did not have any effect on the temperature or degree of mass loss of the scaffolds. Decomposition of the scaffold material above 300°C was the reason behind the loss of mass.

#### 3.3.3. Swelling

**Figure 5.**
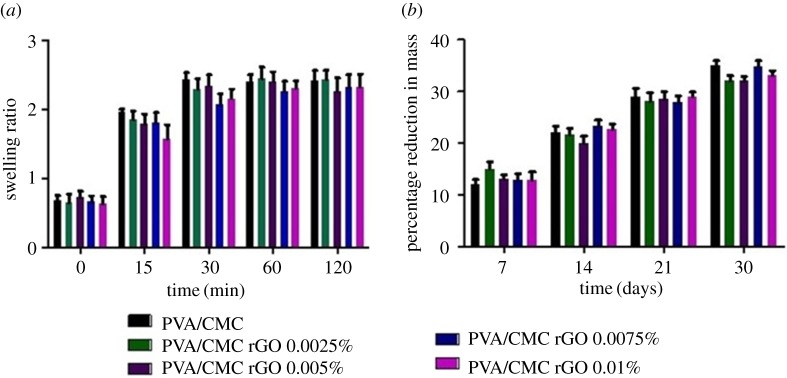
(*a*) Swelling behaviour and (*b*) *in vitro* biodegradation of PVA/CMC, PVA/CMC rGO 0.0025%, PVA/CMC rGO 0.005%, PVA/CMC rGO 0.0075% and PVA/CMC rGO 0.01%.

The mean equilibrium swelling ratio of the scaffolds was approximately 2.3 (figure 5*a*). The differences in swelling ratios of the scaffolds were not significant. Swelling equilibrium was reached within 30 min of immersion in PBS. Incorporation of rGO did not affect the swelling behaviour of the scaffolds.

#### 3.3.4. *In vitro* biodegradation

Degradation of the scaffolds was observed over a period of 30 days in a lysozyme containing PBS solution. The mean mass loss of the scaffolds was approximately 32% at the end of this period (figure 5*b*). No significant difference was observed in the rate or degree of degradation between the scaffolds.

### 3.4. Biological properties of the synthesized scaffolds

#### 3.4.1. Cell viability and attachment on the scaffolds

**Figure 6.**
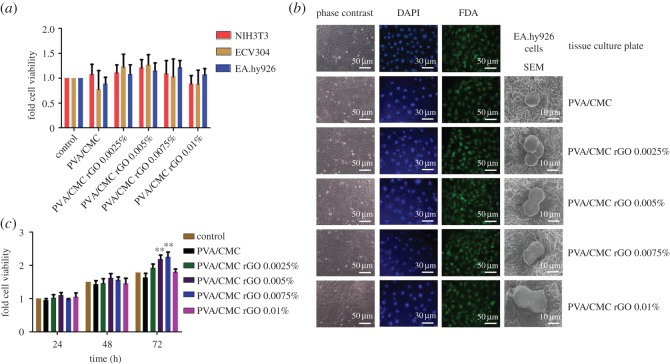
(*a*) Cell viability of NIH3T3, ECV304 and EA.hy926 cultured on the scaffolds, (*b*) phase-contrast (column one), DAPI-stained (column two), FDA-stained (column three) and SEM micrographs (column four) of EA.hy926 cultured on the scaffolds and (*c*) cell proliferation of EA.hy926 cultured on scaffolds up to for 72 h (*n* = 6, **p < 0.01 versus control).

The biocompatible nature of the scaffolds was confirmed via alamarBlue assays performed on three different cell lines: NIH3T3, ECV304 and EA.hy926. The cytotoxicity studies performed by the alamarBlue assay showed that the cell viability of all the three cell lines cultured on rGO-incorporated scaffolds was similar to the cells cultured on the tissue culture plate (control) (figure 6*a*). Recently, it has been shown that rGO is toxic to cells at concentrations above 100 ng ml^−1^ when administered in the form of a solution [31]. Our results show that rGO, when entrapped within a scaffold, is not cytotoxic even at concentrations much higher than its cytotoxic concentration in free solution. The possible reason behind this is the slow rate of release of rGO from the scaffolds into the solution, thereby presenting itself to cells at a concentration lower than the cytotoxic level.

Phase-contrast microscopy of EA.hy926 cells attached onto the scaffolds revealed no significant change in morphology when they are cultured on the rGO-loaded scaffolds (figure 6*b*, first column). DAPI is a fluorescent stain that is used for nuclei staining. DAPI binds to the A–T-rich regions in DNA [48]. Our DAPI-stained images show that the nuclear integrity of the attached cells was maintained (figure 6*b*, second column). The scaffolds absorbed some of the stains and thus had a light blue background which contrasted with the concentrated blue spots corresponding to the nucleus of the cells attached to them.

Cell viability was observed qualitatively in the FDA-stained samples (figure 6*b*, third column). FDA is a non-fluorescent molecule that is taken up by the live cells and hydrolysed to fluorescent fluorescein [49]. As dead cells cannot accumulate FDA and hydrolyse it, only the live cells fluoresce after FDA staining. The fluorescent images obtained after staining cells with FDA (which specifically stains viable cells) corroborated the results obtained in the aforementioned alamarBlue assay. Cells appeared rounded under the scanning electron microscope (figure 6*b*, fourth column). Cells seeded onto the different scaffolds did not show any difference in morphology under SEM. The rounding-up of cells could be attributed to the formaldehyde treatment which was performed to fix the cells onto the scaffolds [50–52].

#### 3.4.2. Endothelial cell proliferation

The alamarBlue assay was performed on EA.hy926 cells cultured on the scaffolds for 24, 48 and 72 h. The results confirmed that the scaffolds did not show any cytotoxicity beyond 24 h as well. Furthermore, after 72 h of cell culture, the cell viability on PVA/CMC rGO 0.005% and PVA/CMC rGO 0.0075% scaffolds were significantly higher than the cell viability on the remaining scaffolds (figure 6*c*). This increased viability indicates an enhanced cell proliferation in these scaffolds. However, the scaffold PVA/CMC rGO 0.01% did not show any increased viability. Hence, the results suggest that cell proliferation increased only up to a certain concentration of rGO.

#### 3.4.3. *In vivo* chick chorioallantoic membrane assay

**Figure 7.**
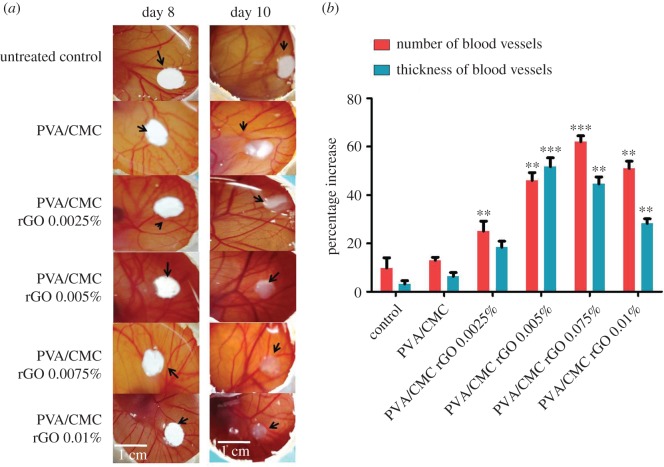
(*a*) Digital images of the untreated and treated CAM and (*b*) percentage increase in the average number of blood vessels and average thickness of blood vessels obtained on day 10 of the CAM assay. The values are normalized to that of the untreated control on day 8 (*n* = 9, **p < 0.01 and ***p < 0.001 versus control).

The CAM assay was performed to evaluate the efficiency of the synthesized scaffolds in causing angiogenesis (figure 7*a*). A significant increase in the number of blood vessels was observed in the PVA/CMC scaffolds incorporated with 0.005, 0.0075 and 0.01% rGO compared with the control (sterile filter paper discs). PVA/CMC rGO 0.0075% showed the highest increase in the number of blood vessels (62%) (figure 7*b*). The PVA/CMC rGO 0.01% showed a 51% increase in the number of vessels. The scaffolds PVA/CMC and PVA/CMC rGO 0.0025% did not show a significant angiogenic activity when compared with the control. Thus, the angiogenic potential of the scaffolds increased with increasing rGO concentration of up to 0.0075%. Beyond 0.0075%, there was a slight decrease in angiogenesis at rGO concentration of 0.01%. The average thickness of the blood vessels also increased over the 2-day period of the assay. The increase in thickness followed a pattern similar to that of the increase in blood vessels. The highest increase in thickness was seen in PVA/CMC rGO 0.005% (51.7%), and PVA/CMC rGO 0.0075% showed a 44.5% increase. PVA/CMC rGO 0.0025% and PVA/CMC rGO 0.01% scaffolds showed 18.4 and 28.2% increase in vessel thickness, respectively (figure 7*b*). The increase in vessel thickness is possibly an outcome of arteriogenesis.

## 4. Discussion

In this study, we have successfully synthesized pro-angiogenic scaffolds by incorporating rGO nanoparticles into PVA/CMC hydrogels. PVA has been used extensively for tissue engineering applications due to its biocompatibility and mechanical strength [34]. Owing to its hydrophilicity, it is usually blended with a natural polymer [34]. Therefore, we used CMC, which is a derivative of a natural polymer, as a copolymer for PVA for developing scaffolds [35,53]. Our molecule of interest, rGO was synthesized from GO and incorporated into the PVA/CMC scaffolds.

rGO was synthesized from GO via sunlight exfoliation method [38,54]. The preparation of rGO from GO via sunlight irradiation bypassed the need for chemical reagents for the reduction process. This rGO was more soluble in water compared to GO, thus resulted in a better dispersion. The rGO and GO dispersions were visually distinguishable, GO displayed a deep brown colour and while rGO dispersion had a black colour.

The presence of functional groups within the rGO made it possible to detect its presence within the scaffolds [55]. As these functional groups interacted non-covalently with the scaffolds, certain peak shifts were observed in Raman spectra of the composite scaffolds when compared to pure PVA/CMC blends. Also, the presence of rGO nanoparticles within PVA/CMC sheets disrupted their crystalline nature and increased the amorphous nature of the scaffolds. The increase in amorphous nature was observed in the XRD spectra, wherein the area under the crystalline peak decreased with an increase in the rGO concentration in the scaffolds. The reason behind the decrease in the degree of crystallinity with increasing rGO concentration could be the destabilizations brought about by the nanoparticles within the repeating regular crystalline PVA/CMC sheets as the nanoparticles took up space within the neighbouring crystal planes. The SEM images show that the scaffolds are uniformly porous. The pores formed within the scaffolds were an outcome of the freezing and lyophilization technique used for developing the scaffolds [56]. The presence of rGO nanoparticles did not significantly affect the rate of freezing or sublimation of the aqueous solvent, thus yielding similar porosity values for all the scaffolds. The porous nature of scaffolds allows the cells seeded on them to penetrate into the scaffolds. Also, the porous nature of the scaffolds helps in supplying enough oxygen and nutrients to the cells and in waste removal.

Incorporation of rGO into the scaffolds did not interfere with the thermal stability of the scaffolds. The melting, glass transition temperatures and decomposition temperature of the scaffolds were not altered. Since rGO does not interact covalently with the PVA/CMC scaffold, the similarity in thermal properties of PVA/CMC and PVA/CMC rGO was expected. The swelling behaviour of the scaffolds roughly resembled a square root of time dependence. Incorporation of rGO did not alter the swelling behaviour of the scaffolds. A change in swelling behaviour is expected when an entity increases the cross-linking density within a hydrogel [57]. As rGO does not interact covalently with either PVA or CMC, the cross-linking density remains the same upon its incorporation into the scaffolds and so does the swelling property. *In vitro* biodegradation of the scaffolds was also not affected due to the incorporation of rGO. The degradation was primarily due to the disruption of glycosidic linkages within the CMC polymeric chains by lysozyme [58]. The low degrees of reduction in mass over 30 days demonstrates the suitability of the synthesized scaffolds for tissue engineering applications.

Cell attachment and viability studies successfully demonstrated the biocompatibility of the scaffolds. Two of the scaffolds were also successful in enhancing the proliferation of EA.hy926 cell line when they were cultured for 72 h, while the other scaffolds retained their biocompatibility over this extended culture period. The increased proliferation could be attributed to the increased period of contact with the rGO, or leaching effect of rGO from degrading scaffolds.

Growth factor release from scaffolds (mostly VEGF) has been the paradigm in the signal-based approach to tackle the issue of enhancing angiogenesis in tissue engineering scaffolds [2]. However, the short half-life of VEGF, or any other protein growth factors, poses a serious problem while determining their efficacy towards the intended purpose [2]. Another problem with the use of protein growth factors is that the traditional scaffolds do not usually control the release rate of the growth factors based on the need for the developing vascular network. This leads to leaky vasculature and formation of poorly developed blood vessels, as they do not get the time to mature due to sustained contact with the growth factors [2]. CAM assay demonstrated the efficacy of our scaffold in angiogenesis. Two days after the scaffolds were implanted on the CAM of a developing chick embryo, angiogenesis was increased significantly compared to the untreated control. When we compared two different parameters that are crucial to angiogenesis in our CAM assay [59], we found an absolute increase in blood vessel thickness along with an increase in the number of blood vessels. The increase in thickness is a sign of the maturation of nascent blood vessels and happens due to a phenomenon called arteriogenesis [59]. Hence, the results obtained suggest that our scaffolds were successful in enhancing both angiogenesis and arteriogenesis. The effect of rGO can be attributed to its ability to increase the intracellular reactive oxygen species concentration, as demonstrated by a recent paper [31]. This increase in reactive oxygen species concentration then triggers the biochemical machinery which is responsible for both angiogenesis and arteriogenesis [31]. Further studies will have to be performed to understand the molecular mechanism behind our observations. In effect, we were able to synthesize nanocomposite scaffolds, incorporated with a non-protein pro-angiogenic entity, which were successful in enhancing angiogenesis in a CAM model. Further studies will have to be carried out to understand the mechanisms that govern the observations.

## Supplementary Material

Raw data for the figures in the manuscript

## Data accessibility

The supporting data are accessible in the electronic supplementary material.
